# IGF-IEc expression is increased in secondary compared to primary foci in neuroendocrine neoplasms

**DOI:** 10.18632/oncotarget.20743

**Published:** 2017-09-08

**Authors:** Krystallenia I. Alexandraki, Anastassios Philippou, Georgios Boutzios, Irini Theohari, Michael Koutsilieris, Ioanna Kassiani Delladetsima, Gregory A. Kaltsas

**Affiliations:** ^1^ Department of Pathophysiology, Medical School, National and Kapodistrian University of Athens, Athens, Greece; ^2^ Department of Experimental Physiology, Medical School, National and Kapodistrian University of Athens, Athens, Greece; ^3^ First Department of Pathology, Medical School, National and Kapodistrian University of Athens, Athens, Greece

**Keywords:** neuroendocrine neoplasms, IGF-IEc, IGF-I, Ki-67, metastasis

## Abstract

Different Insulin-like growth factor-I (IGF-I) mRNA transcripts are produced by alternative splicing and particularly the IGF-IEc isoform has been implicated in the development and/or progression of various types of cancer. In the present study, we examined the potential role of IGF-IEc expression as a new immunohistochemical marker of aggressiveness in neuroendocrine neoplasms (NENs). We utilized immunohistochemical analysis in tissue specimens of 47 patients with NENs, to evaluate the expression of IGF-IEc (%) and Ki-67 proliferation index (%). Specimens from patients with tumors of different tissue origin, of either primary or metastatic lesions and of different grade were examined. Cytoplasmic IGF-IEc staining was found in 23 specimens of NENs or NECs: 10 pancreatic, 4 small bowel, 3 gastric, 1 lung, 1 uterine and 4 poorly differentiated of unknown primary origin. Ki-67 and IGF-IEc expression was positively correlated in all the samples studied (r=0.31, p=0.03). IGF-1Ec expression was more prevalent in specimens originating from metastatic foci with high Ki-67 compared to primary sites with low Ki-67 expression (p=0.036). These findings suggest a possible role of IGF-IEc in NEN tumorigenesis and progression to metastases that could be used as an additional new marker of a more aggressive behavior and a potential drugable target.

## INTRODUCTION

Neuroendocrine neoplasms (NENs) constitute a wide range of tumors derived from neuroendocrine cells that are widely distributed throughout the human body [1]. A distinctive feature of these tumors is their ability to synthesize, and secrete amines and peptides, which when biologically active, may cause distinct clinical syndromes (functional tumors) [2]. Although gastrointestinal NENs (GI-NENs) are in their majority slow growing tumors, a significant number exhibits metastatic disease at diagnosis, making early diagnosis and identification of malignant behavior markers of outmost importance [3].

The insulin-like growth factor (IGF) system is considered to play an important role in GI-NENs [4–6]. Components of the IGF system are the IGF-I and IGF-II, type 1 (IGF-IR) and type 2 (IGF-IIR) IGF receptor, insulin receptor (IR) isoforms A (IR-A) and B (IR-B), and at least six IGF-binding proteins (IGFBP1–6) [7]. Expression of IGFs and their cognate receptors correlate with increased angiogenesis, development of metastases, reduced survival and tumor de-differentiation in several cancer types including NENs [8–11]. IGF-I acts in tissue cell model of pancreatic NENs (pNENs: BON cells) through the IGF-IR, regulating cell-cycle proteins and stimulating cellular secretion of Chromogranin A (CgA) [12–15]. In addition, IGF-II and IGF-IR are also expressed in 30% and 70% of NENs, respectively [8], whereas high IGF-II levels stimulate BON cell growth [15].

A new component of the IGF system has recently been studied for its potential role in carcinogenesis. Specifically, the alternative splicing of the *igf1* gene results in different IGF-I mRNA transcripts encoding several IGF-I precursor proteins (isoforms), such as IGF-IEa, IGF-IEb, and IGF-IEc [16]. These IGF-I precursors undergo post-translational cleavage, generating the common mature IGF-I peptide and different carboxyl-terminal extension (E-) peptides. There is increasing interest for the potential role of IGF-I isoforms and/or their respective non-(mature) IGF-I products in the regulation of distinct biological activities. Differential expression of the IGF-I splice variants in normal versus cancer tissues implies that the expression pattern of the various IGF-I isoforms may possess different functions in cancer biology, particularly the IGF-IEc [16–20].

The aim of the present study was to investigate the level and pattern of the IGF-IEc isoform expression in NENs, in order to investigate its potential role in NENs pathogenesis, regarding to their differentiation state and metastatic behavior.

## RESULTS

### Examined material

The examined specimens included 47 cases of NENs: 8 gastric, 17 pancreatic, 3 appendiceal, 9 small bowel, 1 colonic, 1 retro-sigmoidal, 1 gallbladder, 2 lung and 4 poorly differentiated of unknown primary origin (UPO) (2 with small cells; 2 with large cells), and 1 cervical NEN. Thirty specimens were derived from the primary tumors and 17 from metastatic sites (11 liver biopsies, 4 lymph nodes, and 2 other metastatic foci) (Table 1).

**Table 1:** The surgical specimens and the biopsies studied along with the of IGF-IEc expression (%)

NEN type	N	Ki-67 (%)	Specimens	IGF-IEc expression (%)
Appendiceal NEN (N=3)	1	1	Surgical Specimen	0
	1	2	Surgical Specimen	0
	1	3	Lymph node	0
Colonic NEN	1	2	Surgical Specimen	0
Gallbladder NEN	1	6	Surgical Specimen	0
Gastric NEN (N=8)	3	2	Surgical Specimen	0
	1	5	Surgical specimen	0
	1	85	Surgical Specimen	0
	1	5	Surgical Specimen	10
	1	2	Surgical Specimen	50
	1	2	Surgical Specimen	100
Rectosigmoidal NEN (N=1)	1	50	Surgical Specimen	0
Pancreatic NEN (N=17)	1	1	Surgical Specimen	0
	1	6,50	Surgical Specimen	0
	2	7	Surgical Specimen	0
	1	17	Surgical Specimen	0
	1	50	Surgical Specimen	0
	1	55	Liver biopsy	0
	1	2	Surgical Specimen	1
	1	2.5	Surgical Specimen	1
	1	80	Liver biopsy	1
	1	2	Surgical Specimen	10
	1	3	Surgical Specimen	10
	1	6	Liver biopsy	10
	1	20	Liver biopsy	10
	1	15	Liver biopsy	50
	1	60	Liver biopsy	80
	1	6	Liver biopsy	100
Small bowel NEN (N=9)	2	1	Surgical Specimen	0
	3	1	Liver biopsy	0
	1	2	Surgical Specimen	1
	1	5	Surgical Specimen	1
	1	1,5	Liver biopsy	10
	1	5	Surgical Specimen	10
Lung NEC (N=2)	1	50	Surgical Specimen	0
	1	70	Lymph node	50
Uterine cervical NEC	1	70	Surgical Specimen	10
Undifferentiated NEC (Small cell) UPO	1	25	Bone marrow metastasis	1
	1	80	Lymph node	10
Undifferentiated NEC (Large cells) UPO	1	70	Lymph node	50
	1	70	Metastasis mediastinum	100
Total	47	5 (1-85)	Origin from primary tumors (30)/ metastases (17)	10 (1-100)

NEN: neuroendocrine neoplasm; NEC: neuroendocrine carcinoma; UPO: unknown primary origin.

### Clinical data

Patients’ characteristics, clinical characteristics of the neoplasms (functional or non-functional NENs and positivity or not to somatostatin receptor scintigraphy), pathologic features of neoplasms derived from patients with positive and negative staining for IGF-IEc are shown in Table 2.

**Table 2:** The differences between specimens with positive and negative immune reaction for IGF-IEc, regarding patients’ characteristics and neoplasms features

	All specimens studied	Specimens with IGF-IEc staining	Specimens without IGF-IEc staining	p-value
Number	47	23 (49%)	24 (51%)	
Males (%)	28 (59.6%)	14 (60.9)	14 (58.3)	0.74
Age (years±SD)	55.2±13.3	58.5±12.2	52.1±13.9	0.16
Ki-67 (%) median value (range)	5 (1-85)	6 (2-80)	2.5 (1-85)	**0.055**
SRS, positive/N (% positive)	13/24 (54.2)	5/11 (45.5)	8/13 (61.5)	0.70
Functional syndrome, N (%)	6/26 (23.1)	4/13 (30.8)	2/13 (15.4)	0.65
Specimen taken from primary site (%)	30 (63.8)	11 (47.8)	19 (79.2)	**0.024**
Specimen taken from metastatic site (%)	17 (36.2)	12 (52.2)	5 (20.8)	
G1 (%)	18 (38.3)	6 (26.1)	12 (50)	0.227
G2 (%)	16 (34)	9 (39.1)	7 (29.2)	
G3 (%)	13 (27.7)	8 (34.8)	5 (20.8)	

P value < 0.05; NEN: neuroendocrine neoplasm; G: grade of differentiation per Ki-67, for G1 ≤ 2%, G2 2-20%, G3 > 20%.

### Cytoplasmic IGF-IEc staining in surgical and biopsy specimens (Figure 1)

Cytoplasmic staining was found overall in 23 specimens. Cases with positive IGF-IEc immunohistochemical expression were found in NENs or NECs: 10/17 (58.8%) pancreatic, 4/9 (44.4%) small bowel, 4 (100%) poorly differentiated UPO, 3/8 (37.5%) gastric, 1/2 (50%) lung and 1 uterine cervical (Figure 2). Positive expression was found in 12/17 (70.6%) specimens from metastatic sites and in 11/30 (36.7%) specimens from primary tumors (p=0.024).

**Figure 1:**
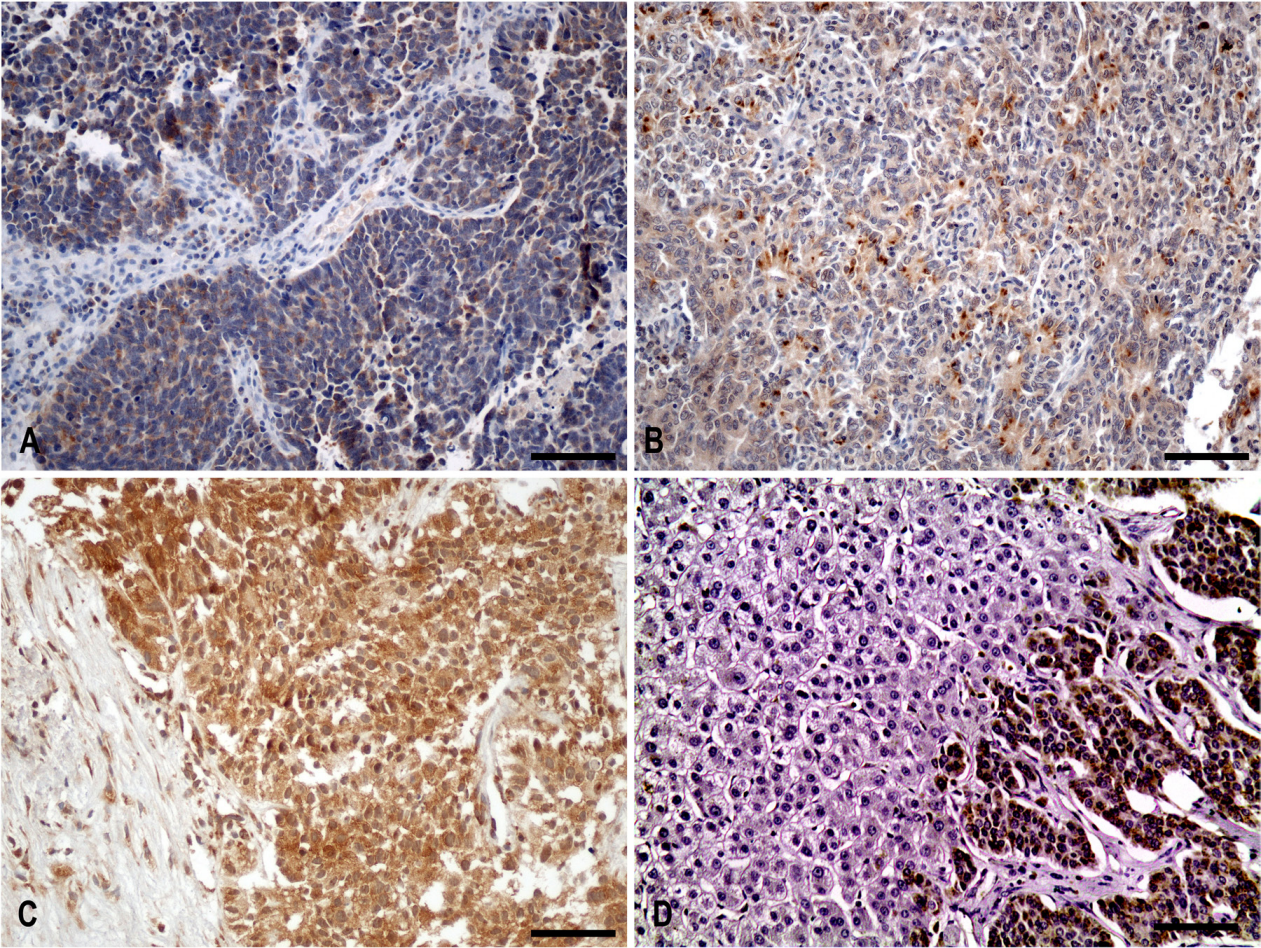
Cytoplasmic IGF-IEc staining in surgical and biopsy specimens **(A)** Diffuse cytoplasmic staining of IGF-IEc expression in metastatic (lymph node) lung neuroendocrine carcinoma (small cell carcinoma) of a 67-year-old male patient (x200, IHC). **(B)** Diffuse and dot-like cytoplasmic staining pattern of IGF-IEc expression in G2 primary small bowel neuroendocrine neoplasm of a 55-year-old male patient (x200, IHC). **(C)** Diffuse cytoplasmic staining of IGF-IEc expression in G3 metastatic pancreatic neuroendocrine carcinoma; liver biopsy of a 69-year-old male patient (x200, IHC). **(D)** Negative hepatocellular expression of IGF-IEc in contrast to the positivity of metastatic tumor cells (x200). Scale bar: 100μm.

**Figure 2:**
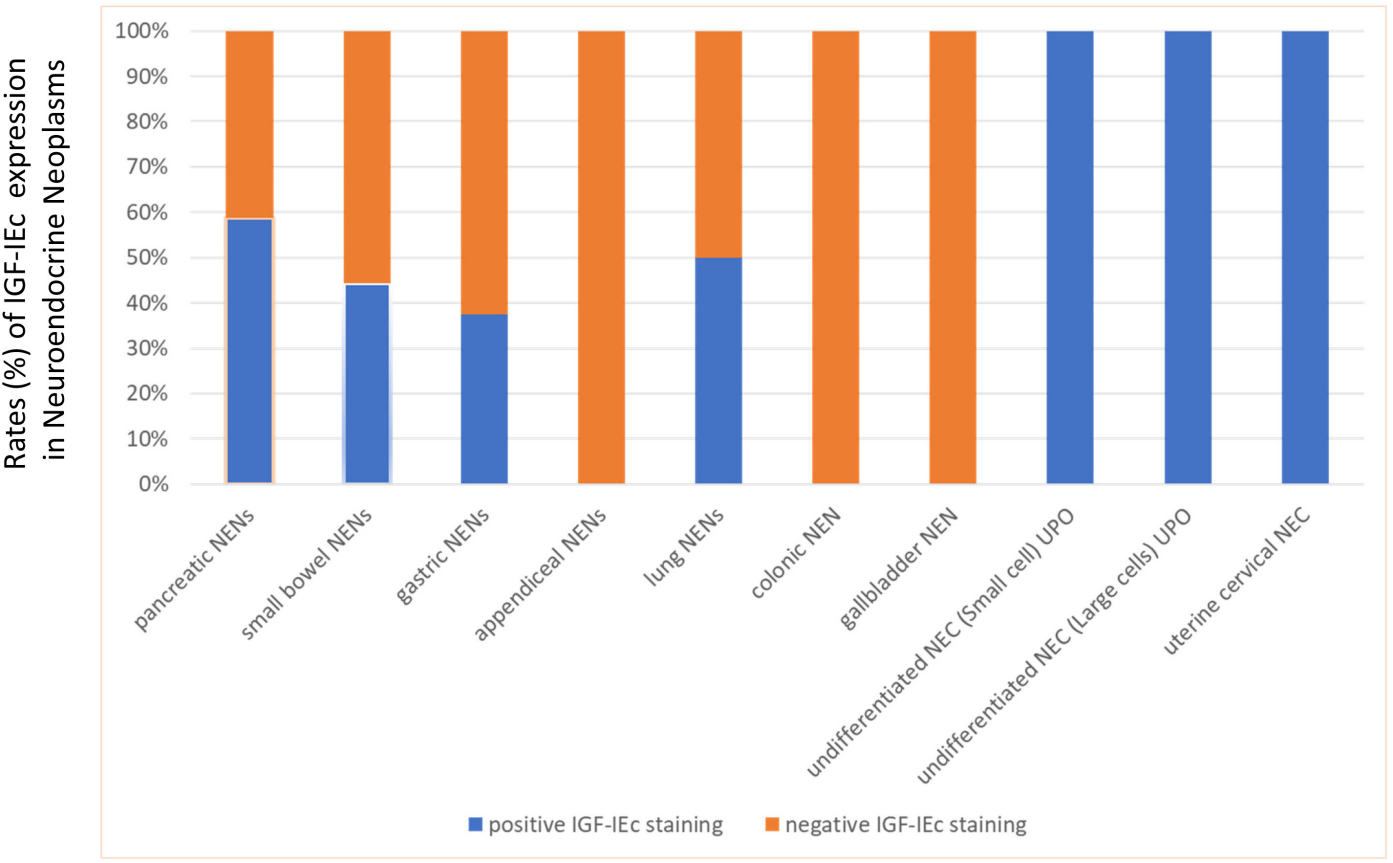
Expression (%) of IGF-IEc in the examined neuroendocrine neoplasms (NENs) Appendiceal, colonic and gallbladder NENs were IGF-IEc negative, while all NEC showed positive immune reaction for IGF-IEc.

### IGF-IEc expression was higher in metastatic sites compared to primary tumors

IGF-IEc expression was more prevalent in specimens derived from metastatic sites compared to specimens from primary tumors and this difference was statistically significant (chi-square= 5.11, p=0.024) (Table 2). Moreover, the samples from metastatic sites had higher immunohistochemical expression of IGF-IEc compared to samples from primary tumors [10 (0-100) versus 0 (0-100), respectively, p=0.03].

Multiple logistic regression analysis showed that metastatic site and age were the best predictors of IGF-IEc expression compared to sex, Ki-67 expression, or grading (Table 3).

**Table 3:** Confounding parameters and the IGF-IEc expression (%)

Factors studied	Results from Multiple Analysis		
	OR	95%CI	*P-value*
Age at the time of biopsy	1.081	1.002-1.166	**0.045**
Gender	2.929	0.536-15.997	0.215
Origin from primary (1) or metastatic (2) site	9.631	1.489-62.283	**0.017**
Ki-67 expression (%)	0.998	0.967-1031	0.911

Multiple logistic regression analysis was used to reveal the best predictor(s) of IGF-IEc expression.

### Ki-67 expression was positively related to the IGF-IEc expression

In the total examined material, IGF-IEc expression was positively correlated with Ki-67 value (r=0.31, p=0.03). Moreover, in the G1 group of NENs, a trend for a positive correlation was found between IGF-IEc expression and the Ki-67 expression (r=0.47, p=0.051).

Finally, Ki-67 expression (%) was negatively correlated with CgA (r=-0.66, p=0.02) and synaptophysin (%) expression (r=-0.50, p=0.03).

## DISCUSSION

This study provides evidence that the alternative splicing of the *igf1* gene results in different IGF-I mRNA transcripts encoding several IGF-I isoforms that could be implicated in the progression and aggressive behavior of GI-NENs. In particular, the IGF-IEc isoform showed higher expression in NENs derived from metastatic sites and in those with poor differentiation.

NENs exhibit significant differences in growth behavior, from very slow to fast growing and aggressive tumors [6, 21]. This heterogeneity may be reflected on differences of the IGF system component expression. The heterogeneity of IGF system in human NENs has been confirmed in a series of 37 tumors including gastrinomas, insulinomas, carcinoids and non-functioning NENs [8, 22]. In another study, using an *in vivo* model of liver metastases from a carcinoid tumor, the serotonin-IGF-I axis was found to act differentially, depending on serotonin levels [23]. Despite the small number of cases examined in our study, our findings provide further support for the heterogeneous expression of IGF-IEc in tumors of different origin and differentiation. Pancreatic and small bowel NENs, being more prevalent in our series, were found to express IGF-IEc approximately in half of the cases compared to none in the less aggressive NENs as those derived from the appendix. Moreover, higher IGF-IEc expression was significantly associated with higher Ki-67 expression. IGF-IEc expression was increased in metastatic sites compared to primary tumors, suggesting that it may be involved in the metastatic process and constituting a marker of aggressive tumor behavior. Multivariate analysis revealed this latter finding, along with age, as the most important predictors of IGF-IEc expression. Despite the paucity of data available, another study did not document any significant correlation between Ki-67 and mRNA expression of IGF system components, but the IGF-IEc variant was not investigated [24].

The IGF-IEc isoform appears to be directly involved with tumorigenesis [17, 19, 25], although the exact mechanism by which it exerts its biological neoplastic effect is currently under investigation. It has been shown that the Ec domain of the IGF-IEc possesses bioactivity, which is possibly mediated via an autonomous, IGF-IR and IR-independent mechanism [6, 26]. A synthetic analogue of the Ec domain has recently been shown to increase growth and metastatic potential in human prostate cancer PC-3 cells [27]. Immunohistochemical analysis from prostate cancer specimens revealed that the IGF-1Ec isoform was positively associated with cancer stage and grade [25]. In parallel with these findings, IGF-IEc was found to be over-expressed in malignant osteosarcoma cells, while it was not expressed in the least malignant cells [19, 28], implying that it exerts its role predominantly in cancer progression [16]. This notion is in line with our finding of the preferential expression of IGF-IEc in less differentiated and metastatic NENs. Interestingly, the effect of anti-IGF-1Ec antibody has been already examined in a prostate cancer cell line with beneficial results [25] and may be of benefit in NENs as well.

Although our study shows for the first time a potential role of the IGF-IEc in GI-NENs, it also has a number of limitations including the relatively small number of cases studied. In addition, the experimental design of our study did not allow the examination of fresh tissue to document the mRNA presence of this isoform.

In conclusion, the preferential expression of IGF-IEc in less differentiated and metastatic NENs provides evidence of its use as a potential marker of tumor aggressiveness or dedifferentiation and a potential drugable target.

## MATERIALS AND METHODS

### Ethical approval

A written informed consent was obtained by all the patients to participate in this study, which was approved by the Ethics Committee of the “Laiko” University Hospital of Athens, and all experimental procedures conformed to the Declaration of Helsinki.

### Immunohistochemistry

For the detection of synaptophysin, CgA and Ki-67 proliferation index the following antibodies were used: anti-Synaptophysin, clone DAK-SYNAP (1:400; Dako, Glostrup, Denmark); anti-Chromogranin A, clone DAK-A3 (1:150; Dako) and anti-Ki-67, clone MIB1 (1:100; Dako).

Evaluation of all immunohistochemical markers was performed by counting at least 2000 tumour cells. Immunohistochemical analysis for IGF-IEc quantification was performed using an IGF-IEc-specific antibody [29] as previously described [30]. The expression of IGF-IEc, Ki-67, synaptophysin and chromogranin A was evaluated semi-quantitatively by defining the percentage of positively stained cells.

Additionally, NENs subgroups have been studied based on Ki-67 cut-off values, as proposed by the World Health Organization (WHO) classification (G1: ≤ 2%, G2: 3%-20%, G3: >20%) [31].

Clinical data available were registered (gender, age, the presence or absence of a functional syndrome and the positivity or not to somatostatin receptor scintigraphy.

### Statistical analysis

The multiple comparisons among the immunohistochemistry groups were performed with the Kruskal-Wallis test, while Mann-Whitney U test was used for comparisons between two groups. The values are presented as medians (range). Statistical significance was set at <0.05. Correlations between variables were evaluated by Spearman’s correlation coefficient and multiple logistic regression analysis was used to reveal the best predictor(s) of IGF-IEc expression. All analyses were performed using Statistical Package for the Social Sciences (SPSS (version 20; Chicago, IL, USA).

## Abbreviations

Neuroendocrine neoplasms: NENs
chromogranin A: CgA
gastrointestinal neuroendocrine neoplasms: GI-NENs
Ki-67 proliferation index: Ki-67
insulin-like growth factor: IGF
type 1 IGF receptor: IGF-IR
type 2 IGF receptor: IGF-IIR
insulin receptor: IR
insulin receptor isoform A: IR-A
insulin receptor isoform B: IR-B
IGF-binding protein: IGFBP
pancreatic NEN: pNEN
unknown primary origin: UPO
